# Transcriptomic, cellular and life-history responses of *Daphnia magna* chronically exposed to benzotriazoles: Endocrine-disrupting potential and molting effects

**DOI:** 10.1371/journal.pone.0171763

**Published:** 2017-02-14

**Authors:** Maeva Giraudo, Mélanie Douville, Guillaume Cottin, Magali Houde

**Affiliations:** 1 Environment and Climate Change Canada, Aquatic Contaminants Research Division, Water Science and Technology Directorate, Montreal, Québec, Canada; 2 Université Paris Descartes, Paris, France; Jinling Institute of Technology, CHINA

## Abstract

Benzotriazoles (BZTs) are ubiquitous aquatic contaminants used in a wide range of industrial and domestic applications from aircraft deicers to dishwasher tablets. Acute toxicity has been reported in aquatic organisms for some of the BZTs but their mode of action remains unknown. The objectives of this study were to evaluate the transcriptomic response of *D*. *magna* exposed to sublethal doses of 1H-benzotriazole (BTR), 5-methyl-1H-benzotriazole (5MeBTR) and 5-chloro-1H-benzotriazole (5ClBTR) using RNA-sequencing and quantitative real-time PCR. Cellular and life-history endpoints (survival, number of neonates, growth) were also investigated. Significant effects on the molting frequency were observed after 21-d exposure to 5MeBTR and 5ClBTR. No effects on molting frequency were observed for BTR but RNA-seq results indicated that this BZT induced the up-regulation of genes coding for cuticular proteins, which could have compensated the molting disruption. Molting in cladocerans is actively controlled by ecdysteroid hormones. Complementary short-term temporal analysis (4- and 8-d exposure) of the transcription of genes related to molting and hormone-mediated processes indicated that the three compounds had specific modes of action. BTR induced the transcription of genes involved in 20-hydroxyecdysone synthesis, which suggests pro-ecdysteroid properties. 5ClBTR exposure induced protein activity and transcriptional levels of chitinase enzymes, associated with an impact on ecdysteroid signaling pathways, which could explain the decrease in molt frequency. Finally, 5MeBTR seemed to increase molt frequency through epigenetic processes. Overall, results suggested that molting effects observed at the physiological level could be linked to endocrine regulation impacts of BZTs at the molecular level.

## Introduction

Benzotriazoles (BZTs) are a family of high production volume (HPV) chemicals [1] that are used in a broad range of industrial, domestic, and commercial applications and products. The parent compound 1H-benzotriazole (BTR) and its two derivatives 5-methyl-1H-benzotriazole (5MeBTR) and 5-chloro-1H-benzotriazole (5ClBTR) are the most widely employed BZTs [2,3]. BTR and 5MeBTR have metal complexing properties and are used as anticorrosive additives (e.g., in lubricants, waxes, polishes, cooling and hydraulic fluids) and in aircraft deicer and anti-icer fluids [4,5,6], while 5ClBTR is mostly used in photofinishing operations to improve photographic image quality and for ultraviolet light stabilization in plastics [2,7]. In addition, BZTs can serve as chemical intermediate in the production of dyes, pharmaceuticals and fungicides [8,9], can be used in dishwasher reagents for silver protection [10] and some can also be included in pesticides and herbicides [11]. An estimated production of 9000 t/year has been documented in the US in 2004 for all BZTs [2,12] and from the most recent data in the USEPA Chemical Data Reporting (CDR) database, 850 t of BTR was used in the US in 2012; no data were available for 5MeBTR and 5ClBTR [13].

**Fig 1:**
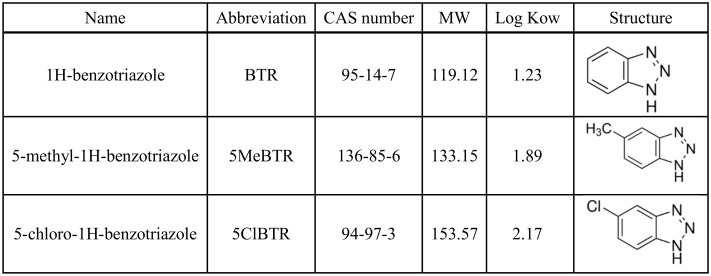
Characteristics of the three benzotriazoles used in this study. Log *K*ow represent octonaol-water partition coefficients from Hart et al. [2].

BZTs are characterized by a low vapor pressure, high water solubility, high polarity, and low octanol-water partition coefficient (log Kow: 1.23 to 2.17; Fig 1) [2], which confers mobility in the aqueous environment. BZTs have been detected ubiquitously in raw and treated wastewaters as well as in surface and ground waters, as recently reviewed in Herrero et al. [4], Cantwell et al. [7] and Careghini et al. [11] (Table 1). Moreover, BZTs are resistant to photochemical and biological degradation, have limited sorption tendency, and are only partially removed by conventional wastewater treatments [2,5,12]; wastewater treatment plants (WWTPs) are therefore one of the most important sources of BZTs into aquatic environments [14,15,16]. BTR is the most commonly detected BZT with concentrations reaching up to 100 μg/L in wastewater effluents [17] and 5.4 μg/L in surface water of the Glatt River in Switzerland [6] (Table 1). 5MeBTR has been reported at lower concentrations of 0 to 200 ng/L in lakes and rivers (Table 1), with one particular occurrence of 2.4 μg/L detected during a survey of 139 streams across the US [18]. Due to its narrower range of use, 5ClBTR has been less studied and measured, and the only concentrations reported in WWTP effluents were lower than the two other BZTs (<260 ng/L) and in the nanogram range in surface waters with one sample from the Netherlands reaching up to 1.5 μg/L [19] (Table 1). In addition, BZTs have been detected in drinking water in the Netherlands and the UK, and human urine samples from seven countries (i.e., US, Greece, Vietnam, Korea, Japan, China, and India), indicating human exposure [9,10,19].

**Table 1:** Environmental water concentrations (in μg/L) reported for BTR, 5MeBTR and 5ClBTR.

Water compartment	BTR	5MeBTR	5ClBTR	References
Airport sampling^a^	126 000	820–17 000		[20,21]
WWTP influents	0.022–44	0.0065–4.9		[3,22,23,24,25]
WWTP effluents	0.01–100	0.002–1.538	0–0.0772	[10,12,15,17,19,22,23,24,25]
Surface water	0.011–5.44	0.02–2.4	0.002–1.5	[3,6,12,17,18,19,25,26,27,28,29,30,31]
Drinking water	0.00006–0.0794	0.01	0.00005–0.0698	[10,19]

^a^ airport groundwater sampling wells and perched water monitoring wells.

BZTs have not yet been reported in tissues of aquatic organisms from impacted environments except for one 54-d in stream exposure study where 5MeBTR was measured in tissue of fathead minnows (*Pimephales promelas*) caged downstream of airport effluent outfalls receiving aircraft deicer and anti-icer fluids [32]. Low bioconcentration factors have been measured for BTR in resting eggs from the freshwater invertebrate *Daphnia longispina-galeata* exposed *in vitro* [33]. However, the high water solubility for these substances would suggest their bioavailability [7].

Toxicity studies on BZTs are scarce in the literature and concern mostly acute toxicity exposures with lethal or inhibition endpoints. BTR and 5MeBTR were found to be toxic at the mg/L level in different aquatic species including freshwater invertebrates from the Daphniidae family [34,35,36], fish species such as fathead minnows (*Pimephales promelas)* and zebrafish (*Danio rerio*) [35,36], the luminescent bacteria *Vibrio fischeri* [32,36,37] and aquatic plants [32,34]. No toxicity data were found for 5ClBTR.

Very little is known for chronic sublethal effects of BZTs and their modes of action in exposed organisms. BZTs are therefore being considered as a prioritized emerging contaminant group for ecotoxicological assessment under the Canadian’s government Chemicals Management Plan [38]. BTR showed anti-estrogenic properties *in vitro* at 1 mg/L using a recombinant yeast assay but no effects were observed on the vitellogenin (VTG) protein expression level *in vivo* in the plasma of fathead minnows exposed for two weeks at the same concentration [39]. In another study however, VTG mRNA levels were increased in the liver, gills and intestines of both males and females medaka (*Oryzias latipes*) exposed for 35-d to 0.01–10 mg/L of BTR, along with the increased transcription of the cytochrome P450 CYP19a in female ovaries, suggesting estrogenic activity [40]. These results indicate an endocrine disruption potential of BTR, which underlines the need for further assessment and rigorous investigation of the chronic toxicity and modes of action of BZTs in aquatic organisms [10,39]. The present study was designed to evaluate the toxicity and better understand the modes of action of BTR, 5MeBTR and 5ClBTR in a model aquatic species, the crustacean *Daphnia magna*.

Recent advances in molecular biology have allowed the development of ecotoxicogenomic tools to measure gene transcription profiles for understanding the mode of action of environmental contaminants and identify biomarkers of exposure and adverse effects [41,42]. Daphnids are good candidate for gene transcription studies due to their parthenogenetic reproduction and have been used to measure the effects of various chemical substances such as metals, pharmaceuticals and flame retardants using cDNA microarrays [41,43,44]. However, microarrays present a certain number of drawbacks including the indirect measurement of transcript abundance depending on hybridization efficiency and the necessity of pre-existing knowledge of the nucleotide sequences spotted on the array [45,46]. The development of high-throughput, next-generation sequencing (NGS) technologies such as RNA-sequencing (RNA-seq) has allowed circumventing these limitations by directly sequencing all cDNA transcripts present in a sample at a given time. RNA-seq represents a more sensitive and specific tool to conduct whole transcriptome profiling in non-model species of interest [47,48] and allows the unbiased detection of novel transcripts.

The objectives of this study were to evaluate the chronic toxicity (21-d) of sublethal concentrations of BTR, 5MeBTR and 5ClBTR in *Daphnia magna* using RNA-seq to measure transcriptomic responses. Further quantitative measurement of gene transcription was realized on a suite of candidate genes to validate the high-throughput results and to evaluate the early gene response of *D*. *magna* (4 and 8-d) to BZT exposure. The transcriptional response was linked to biochemical effects at the protein level and to life-history endpoints (i.e., growth and reproduction) to get a better understanding of the mode of action of BZTs.

## Materials and methods

### *Daphnia magna* culture maintenance and exposure

Genetically homogenous *D*. *magna* were obtained from Quebec Laboratory for Environmental Testing of Environment Canada (Qc, Canada) and cultured in growth chamber following Environment Canada’s method [49]. Cultures were kept at 20±1°C with a photoperiod of 16hr lightness-8hr darkness. Organisms were cultured in Moderately Hard Reconstituted Water (MHRW) [49] and fed every day with green algae *Pseudokirchneriella subcapitata* (3.85×10^5^ cells/mL) and YCT preparation (yeast-cerophyll-trout chow, 0.0125 g/L). All experiments were performed under the same constant temperature and light conditions.

#### Acute toxicity assay

Acute toxicity of BTR, 5MeBTR and 5ClBTR on *D*. *magna* was assessed following Environment and Climate Change Canada test method [49]. Ten neonates (<24-h) were exposed for 48-h without feeding to increasing concentrations of test solution made in MHRW. No solvent was used for BZT solution preparation due to proper water solubility. Endpoints of death were monitored at the end of two individual acute exposures. The LC_50_ was estimated by the Spearman–Karber method (ToxStats, USEPA software).

#### Chronic toxicity assay

Five replicate groups of 12 *D*. *magna* neonates (<24-h) were exposed to two sublethal concentrations of BTR, 5MeBTR and 5ClBTR for 21-d following OECD guidelines [50]. The lowest dose of 2 μg/L was based on the range of environmental concentrations reported in surface waters worldwide and measured in surface water samples from the Hamilton harbor in Lake Ontario, Canada [31]. The higher dose of 2 mg/L corresponds to 1000 × the environmental concentration and falls below the 1/10^th^ of the lowest measured LC_50_ (28.73 mg/L for 5ClBTR; Table 2). Culture medium was used as a control group. New stock solutions were used at every 48-h media renewal where water temperature, conductivity, dissolved oxygen, pH, and hardness were monitored. Spiked culture media and stock solutions were analyzed at the start of the exposure (T0-h) and after 48-h to 96-h of the 21-d chronic exposure experiment to evaluate the chemical stability of BZTs between media renewals (see S1 Protocol). The number of offspring was counted and compared between treatments using a Poisson regression with ordinal model (JMP 9.0.0, SAS Institute Inc.). Body length (n = 6–16/treatment) was defined as the distance from the upper edge of the compound eye to the base of the tail spine and evaluated using a digital image analyzing system (Leica M165c Stereo microscope, Wetzlar, Germany). A significant difference in body length across treatments was tested using ANOVA and Tukey’s HSD test (JMP 9.0.0, SAS Institute Inc.). Organisms used for growth measurements were not used for biochemical or transcriptomic analysis. The number of molts was determined each day and the total number of molts after 21-d of exposure was compared between experimental conditions using ANOVA and Tukey’s HSD test (JMP 9.0.0, SAS Institute Inc.). For each replicate, individual pools of 2–3 individuals were adequately stored at −80°C at the end of the 21-d exposure for further transcriptomic and enzyme activity analyses.

**Table 2:** Acute toxicity values for BTR, 5MeBTR and 5ClBTR in aquatic organisms.

Compound	Species	Endpoint^a^	Results^b^	Reference
BTR	Zooplankton			
	***Daphnia magna***	**48-h LC**_**50**_	**93.3 (76.3–110.3)**	**this study**^c^
	*D*.*magna*	48-h EC_50_	107 (97.1–119)	[34]
	*D*. *magna*	48-h EC_50_	155.4 (154.4–156.5)	[35]
	*D*.*magna*	21-d EC_10_	no effect	[34]
	*Cerodaphnia dubia*	48-h LC_50_	102 (86–120)	[36]
	*Daphnia galeata*	48-h EC_50_	15.8 (13.6–18.3)	[34]
	*D*.*galeata*	21-d EC_10_	0.97 (0.35–2.70)	[34]
	Fish			
	*Pimephales promelas*	96-h LC_50_	65 (38–75)	[36]
	*Danio rerio*	72-h EC_50_	6.43	[35]
	Algae and plant			
	*Desmodesmus subspicatus*	72-h EC_10_	1.18 (0.4–3.49)	[34]
	*Lemna minor*	7-d EC_10_	3.94 (1.95–7.98)	[34]
5MeBTR	Zooplankton			
	***D*. *magna***	**48-h LC**_**50**_	**50.89 (43.58–58.2)**	**this study**
	*D*. *magna*	48-h EC_50_	51.6 (49.7–53.6)	[34]
	*D*. *magna*	21-d EC_10_	5.93 (3.3–10.7)	[34]
	*C*. *dubia*	48-h LC_50_	79 (69–91)	[36]
	*C*. *dubia*	48-h LC_50_	81.3 (70.3–95.1)	[32,37]
	*C*. *dubia*	48-h LC_50_	18–109 (15–137)	[14]
	*D*. *galeata*	48-h EC_50_	5.58 (7.71–9.55)	[34]
	*D*. *galeata*	21-d EC_10_	0.4 (0.08–1.95)	[34]
	Bacteria			
	*Vibrio fischeri*	15-min EC_50_	8.7 (8.2–9.2)	[36]
	*V*. *fischeri*	15-min EC_50_	4.25 (4.18–4.35)	[32,37]
	*V*. *fischeri*	15-min EC_50_	6–8 (5–11)	[14]
	Fish			
	*P*. *promelas*	96-h LC_50_	22 (18–26)	[36]
	*P*. *promelas*	96-h LC_50_	22 (20.5–23.5)	[32,37]
	*P*. *promelas*	96-h LC_50_	8–65 (8–95)	[14]
	Algae and plants			
	*Scelenastrum capricornutum*	96-h IC_25_	23.2 (22–24.7)	[32]
	*D*. *subspicatus*	72-h EC_10_	2.86 (1.68–4.85)	[34]
	*L*. *minor*	7-d EC_10_	2.11 (0.29–14.9)	[34]
5ClBTR	***D*. *magna***	**48-h LC**_**50**_	**28.73 (27.6–29.86)**	**this study**

^a^ EC_x_: Effective Concentration for x% of the organisms, LC_x_: Lethal Concentration for x% of the organisms, IC_x_: inhibition concentration for x% of the organisms.

^b^ Results are expressed in mg/L with confidence intervals in parentheses.

^c^ in bold: 48-h LC_50_ values obtained in this study for *D*. *magna*.

#### Early response assay

Five replicate groups of 12 *D*. *magna* neonates (<24-h) were exposed to 2 mg/L of BTR, 5MeBTR and 5ClBTR for 8-d following the protocol described in the chronic toxicity assay. Culture medium was used as a control group. For each replicate, pools of 3–6 individuals were sampled after 4- and 8-d and adequately stored at −80°C for further gene transcription analyses.

### RNA extraction

Total RNA extractions were performed on the pooled daphnids for each of the 5 independent biological replicates at each time point (4-, 8- and 21-d) using RNeasy^®^plus mini kit (QIAGEN, ON, Canada) following manufacturer’s instructions. For the chronic exposure, one sample of extracted RNA from the highest dose exposure (2 mg/L) and from control organisms was used for RNA-sequencing (N = 4 replicates). Distinct RNA samples from low (2 μg/L) and high (2 mg/L) doses and from control organisms were used for qRT-PCR analysis (N = 5 replicates). RNA was quantified with a NanoDrop™ ND-2000 spectrophotometer (Thermo Fisher Scientific, Mississauga, ON, Canada). RNA integrity and purity was evaluated using a Bio-Rad Experion™ Electrophoresis Station and the RNA StdSens Analysis Kit as per manufacturer's protocols (Bio-Rad, Mississauga, ON, Canada).

### RNA-sequencing

#### cDNA libraries preparation, sequencing and de novo transcriptome assembly

For each of the BZTs (BTR, 5MeBTR, 5ClBTR), 4 biological replicates were used for each treatment condition (control and 2 mg/L BZT-exposure; N = 24 samples) for genomic analyses after 21-d of exposure. cDNA libraries were generated from 250 ng of each RNA samples using the TruseqTM Stranded mRNA sample prep kit (Illumina) following manufacturer’s instructions. The 24 individually tagged libraries were randomly pooled in equal amounts and sequenced on 3 lanes at the McGill University and Genome Quebec Innovation Centre (Montreal, QC, Canada). Multiplex sequencing of 100 paired-end (PE) reads was performed on the Illumina HiSeq2000 instrument. The RNA-seq reads are available through NCBI’s Sequence Read Archive (SRA) under the accession number SRP076999. Reads were trimmed from the 3' end to have a phred score of at least 30. Illumina sequencing adapters were removed from the reads, and all reads had a minimum length of 50 bp. Trimming and clipping were performed using Trimmomatic [51]. Data were normalized by reducing the number of reads using the Trinity normalization utility inspired by the Diginorm algorithm [52]. *De novo* assembly of the transcriptome was realized following the protocol described in [53] and using the Trinity assembler software suite [54].

#### Differential gene transcription analysis and annotation

Gene abundance estimation was performed using RSEM (RNA-Seq by Expectation Maximization) [55] and differential gene transcription analysis was done using the DESeq Bioconductor package in R [56]. Fold changes (FC) in abundance of transcripts in *D*. *magna* exposed to 2 mg/L of BTR, 5MeBTR and 5ClBTR were determined relative to control individuals exposed to culture medium only. Based on the negative binomial distribution implemented in DESeq, only transcripts whose abundance was significantly (*p*<0.05) 4-fold greater or lesser than in the control samples (i.e. absolute log_2_FC of 2) were treated as differentially transcribed genes for each experimental condition.

Differentially transcribed genes were annotated by retrieving the closest protein homolog annotation using translated BLAST searches (blastx– http://blast.ncbi.nlm.nih.gov/Blast.cgi) restricted to the arthropod database and with an e-value cut-off of 1e-05. Sequences homologous to unknown proteins or without known homologues were further annotated by blastn searches in the interactive *D*. *magna* draft genome database [57] available at wFleaBase.org [58]. For transcripts with the same name, if the blastn hits from the draft *D*. *magna* genome resulted in different isoforms of the same geneID, then only the longest transcript with the best annotation e-value was conserved. All genes were additionally submitted to a thorough bibliographic search for functional classification.

### Quantitative real-time PCR (qRT-PCR)

Quantitative RT-PCR analyses were conducted to validate RNA-sequencing data for selected transcripts and to study BZTs mode of action for a suite of candidate genes involved in molting and other endocrine-mediated processes. Total RNA (1 μg) was reverse transcribed using the QuantiTect^®^ Reverse transcription kit (QIAGEN, Toronto, On, Canada) following manufacturer’s instructions. qRT-PCR analyses were then carried out on a CFX96 Touch^™^ real-time PCR detection system using iQ SYBR green Supermix (Bio-Rad) with a final concentration of 300 nM for each primer in a total reaction volume of 13 μL. The PCR conditions were as follows: 95°C for 2 min, followed by 40 cycles of 95°C for 15 s, 60°C for 15 s, and 68°C for 15 s. Primers were either retrieved from published sequences when available or designed using Primer-BLAST (http://www.ncbi.nlm.nih.gov/tools/primer-blast/). Name and symbol of genes as well as primer-specific efficiency and sequence are listed in S1 Table. Each reaction was run in technical duplicate and the mean of five independent biological replicates was calculated. All results were normalized using mRNA level of three reference genes (*tbp*, *ub* and *gapdh*) and relative transcription values were calculated in R using an in-house qPCR analysis package based on the qBase relative quantification software [59] and developed by the Sophia Agrobiotech Institute (INRA Sophia-Antipolis, France) as detailed in Hilliou and Tran [60].

### Chitinase activity

Chitinase activity was measured in *D*. *magna* exposed for 21-d to 2 and 2000 μg/L of BTR, 5MeBTR and 5ClBTR based on published methods [61,62]. Homogenates were centrifuged at 10 000 x g, for 3 min at 4°C. Five μL of S10 was added to 45 μL of substrate solution containing 1.5 mM chitobioside (NPDC) in 0.15 M citrate-phosphate buffer pH 5.5, and incubated with agitation at 37°C for 45 min. Reaction was stopped with 100 μL 0.5 N NaOH. Absorbance was read at 405 nm and results were estimated with a standard curve of 4-nitrophenol (0.48, 0.96, 1.9, 3.9, and 7.7 μM) and expressed as the mean of 5 independent biological replicates in μM 4-nitrophenol/min/mg protein. A significant difference in chitinase activity across treatments was tested using Kruskal-Wallis one-way analysis of variance (JMP 10.0.0, SAS Institute Inc.).

## Results and discussion

### Acute and chronic toxicity

The 48-h acute toxicity testing of BZTs resulted in the mortality of 50% individuals (LC_50_) at 93.3 mg/L for BTR, 50.89 mg/L for 5MeBTR and 28.73 mg/L for 5ClBTR (Table 2). These results are in the same ranges than concentrations causing the immobilization of 50% of the individuals (i.e., EC_50_) reported in *D*. *magna* for BTR and 5MeBTR (Table 2). The LC_50_ value observed for 5ClBTR was used to determine chronic exposure concentrations for the three BZTs.

Chemical analyses during the chronic exposure showed that BZTs concentrations remained stable between media renewals (0.75–2900 μg/L with 7–17% of standard deviation), ensuring continuous exposure of organisms to accurate doses (S2 Table). The variability observed between sampling times is in line with a previous biotransformation study, which reported an increase in the concentration of BTR and 5MeBTR in the first 24-h in the control condition [63].

**Fig 2:**
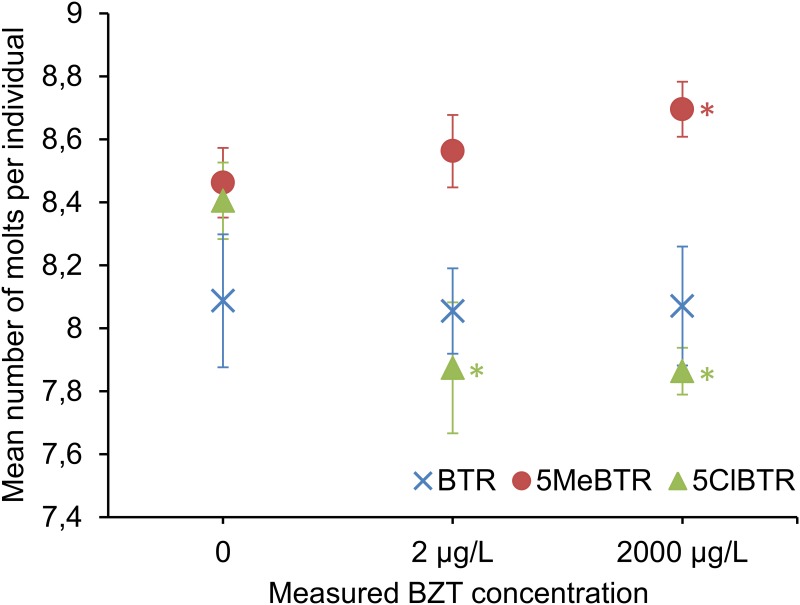
Mean number of molts per individual *D*. *magna* following 21-d exposure to 0, 2 μg/L and 2 mg/L of BTR, 5MeBTR and 5ClBTR. * indicates a significant difference compared to the corresponding control.

Chronic 21-d exposure to sublethal concentrations of 2 μg/L and 2 mg/L of BTR, 5MeBTR and 5ClBTR did not impact the growth of *D*. *magna*, as measured by body length, nor did it affect the total number of neonates produced over a period of 21-d. The frequency of molting was not impacted by BTR but was significantly altered by both 5MeBTR and 5ClBTR. 5MeBTR significantly increased the molting frequency in *D*. *magna* after 21-d exposure to 2 mg/L compared to unexposed controls, whereas 5ClBTR chronic exposure resulted in a significant decreased number of molts in response to both concentrations of 2 μg/L and 2 mg/L (Fig 2).

Molting is an important physiological process for crustaceans during which they shed their old exoskeleton for a new larger cuticle in order to allow growth and development [64,65]. The adverse effects of environmental contaminants on crustacean molting were first described in the 1970s and have since then been reported for over 20 chemicals [66]. In *D*. *magna*, pharmaceuticals [67,68], pesticides [65,69–71], polybrominated diphenyl ethers (PBDEs) [66], polychlorinated biphenyls (PCBs) [65], and xenoestrogens [72, 73] have all shown inhibitory effects on molting. The mechanisms by which these chemicals alter molting in cladocerans are still largely unknown, but may potentially reflect disruption of the endocrine control of molting [66]. The molting process in crustaceans is regulated by a multihormonal system, which is under immediate control of molt-promoting steroid hormones, called ecdysteroids [74]. Similarly to arthropods, the ecdysteroid 20-hydroxyecdysone (20HE) is the main molting hormone in *D*. *magna* [64]. Alterations in molt frequency can be highly indicative of disruption of normal ecdysteroid signaling [75,76]. Anti-ecdysteroids in crustaceans can work as 20HE synthesis inhibitors but most often act as antagonists of the ecdysteroid receptor [70,77]. Indeed, structural similarities between anti-ecdysteroid compounds and endogenous hormones allow the binding and blocking of ecdysteroid receptor, preventing the action of naturally-occurring ecdysteroids, thereby resulting in a slowing of the molting process [65]. For instance, exposure of *D*. *magna* to testosterone and endosulfan sulfate delayed molting, which could be restored by the co-exposure to 20HE, indicating that these compounds acted as anti-ecdysteroids [71,78].

The decreased frequency of molts observed in the present study in response to 5ClBTR might suggest that this chemical has anti-ecdysteroid properties in *D*. *magna* by interacting with the ecdysteroid receptor. This affinity might be explained by the presence of an *ortho*-chlorine on the benzene ring; it has been shown that PCB congeners with *ortho*- and *para*-chlorine substitutions have a strong affinity to the estrogen receptor [79]. On another hand, 5ClBTR might also act as an agonist of the ecdysone receptor that could result in the decreased number of molts. Molting is induced by increased concentrations of 20HE followed by a drop back to basal levels, which triggers ecdysis [75]. 5ClBTR might therefore act as an ecdysteroid-mimic, which may override the typical drop of 20HE levels just prior to exuviation, resulting in molting impairment. Both agonist and antagonist hypotheses of 5ClBTR need further analyses to be confirmed, such as co-exposure to 20HE.

The stimulation of molting by endocrine disruptors, as observed here for 5MeBTR, has only been reported in a few studies on decapod crustaceans and resulted in premature molting or shorter intermolt periods rather than an increased frequency. For instance, the pesticide emamectin benzoate induced premature molting in the American lobster *Homarus americanus* by interfering with the Molt-Inhibiting Hormone (MIH) [80], which has not been reported in *D*. *magna* [81]. One occurrence of an increased number of molts was reported in *D*. *magna* in response to ponasterone A, an ecdysteroid found in plants [82]. However, these experiments did not provide mechanistic support for the ecdysteroidal action of ponasterone A [75]. The observed increase of the number of molts in *D*. *magna* in response to 5MeBTR is therefore difficult to explain based solely on the reported ecdysteroid-mediated effects and needs further investigation.

Overall, these results strongly suggest that 5MeBTR and 5ClBTR may have endocrine disruption potential in *D*. *magna* at sublethal levels. Further measurement of the transcriptional response to these BZTs will help identify the potential pathways involved.

### RNA-seq *de novo* assembly

Transcriptome sequencing was performed using an Illumina HiSeq2000 sequencer for 24 libraries from *D*. *magna* exposed to 0 or 2 mg/L of BTR, 5MeBTR and 5ClBTR. The transcriptome assembly produced a total of 629,397,113 clean paired reads after quality filtering and removing of low quality reads (S3 Table). Using the Trinity assembly program, a total of 41,538 putative transcripts clustered into 14,666 components was generated, with a mean length of 2,385 bp and 50% of the assembly were contained in transcripts larger than 3,200 bp (N50 = 3,263) (S4 Table). These numbers are consistent with a recent study in *D*. *pulex*, suggesting the robustness of the present transcriptome data [83].

### Differential gene transcription analysis

**Fig 3:**
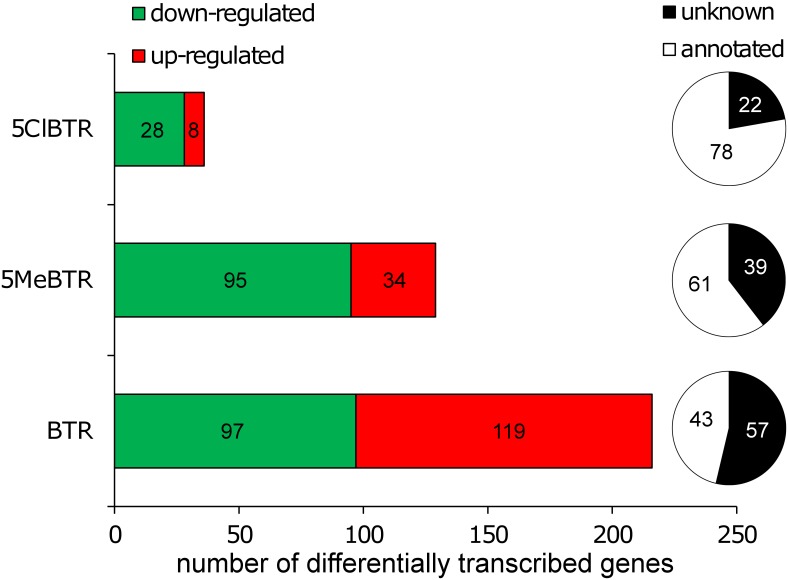
Number of significantly up- and down-regulated genes by 21-d exposure to 2 mg/L of BTR, 5MeBTR and 5ClBTR in *D*.*magna* measured by RNA-seq (logFC±2, *p*<0.05). Pie charts indicate the percentage of differentially transcribed genes with and without a predicted function from blastx annotation searches.

The abundance of constructed transcripts was compared between exposed and control samples using DESeq to identify differentially transcribed genes (log_2_FC±2, *p*<0.05). The list of all differentially transcribed genes with their predicted function and corresponding log_2_ transcription ratios for each treatment are given in the Supplementary Information (S5 Table). Results indicated that individual exposure to the three BZTs impacted the transcription of a total of 381 genes, and that more than 45% of them could be associated with a potential function following successive annotation steps (Fig 3). Annotated transcripts were grouped into different functional categories based on bibliographic searches (Table 3). The major biological pathways affected by BZT exposure at the transcriptomic level were molting, development and 20HE-mediated processes, which would corroborate the molting frequency effects observed and the endocrine disruption potential of these chemicals. Although similar pathways were affected by all BZTs, there were no genes commonly impacted by 5ClBTR and the two other BZTs and only 17 genes were affected both by BTR and 5MeBTR (S1 Fig). These results suggest specific modes of action for each of these compounds and may explain the different effects on molt frequency observed for each BZT.

**Table 3:** Number of annotated up- and down-regulated genes measured by RNA-sequencing in response to 2 mg/L exposure to BTR, 5MeBTR and 5ClBTR.

	BTR		5MeBTR		5ClBTR	
	up	down	up	down	up	down
**molting**^a^	**14**	**3**		**5**	**1**	
**20E**	**6**		**1**	**1**		**1**
**development / cell morphogenesis**	**5**	**3**	**2**	**12**		**1**
glycan	1	2		3		1
lipid metabolism	2	7	1	2	1	2
structural proteins		4		1		
protein metabolism	8	4	1	4		2
energy metabolism	1	2		1		
retinol metabolism			1			
RNA processing and metabolism	2	1		1		
transcription/translation	3	4	1	4		1
cytoskeleton	2			3		
oxidative stress	2	1			1	
ion transport, homeostasis	6		2	1		
Membrane trafficking			1		1	
response to drug				2		
immune response		1	1			
other functions	11	7	4	8		

^a^ In bold are the most impacted pathways (i.e., highest number of differentially expressed genes) related to molting and developmental processes.

**Fig 4:**
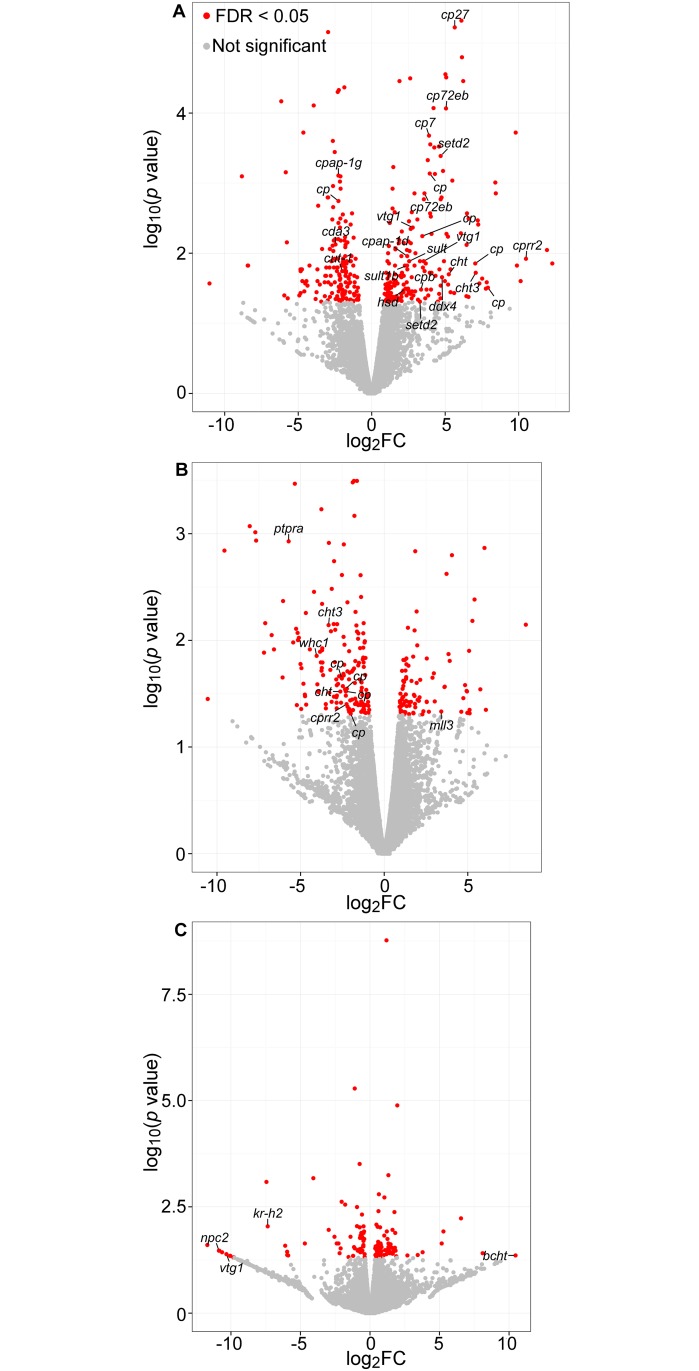
RNAseq data showing up- and down-regulated genes (x axis represents positive and negative fold changes, respectively) in *D*. *magna* exposed to 2 mg/L of (A) BTR, (B) 5MeBTR and (C) 5ClBTR. Genes highlighted in red are significantly differentially transcribed between exposed and control samples (log_2_FC ± 2, *p*<0.05). Annotated genes with a predicted function related to molting and 20HE-mediated processes are indicated. Acronym definition can be found in S6 Table.

BTR had the most potent effect on gene transcription by inducing the up-regulation of 119 genes, including 20 genes related to molting and ecdysteroid-mediated processes (Table 3, S5 Table). Nine genes coding for cuticular proteins were among the most significantly up-regulated genes (Fig 4A). Daphnia exoskeleton, or cuticle, is made primarily of an assembly of chitin and cuticular proteins [84,85]. During molting, shedding of the old cuticle and synthesis of the new one are directly controlled by ecdysteroids titers [86]. In *D*. *magna*, numerous cuticle proteins coding genes were found significantly induced in response to 20HE and repressed by the anti-ecdysteroid fenarimol [42]. In subsequent studies, fenoxycarb, a juvenile hormone agonist (JHA) with anti-ecdysteroid activity, was found to both increase and decrease cuticle genes mRNA levels [87,88]. Similar observations were made for another JHA, epofenonane [87]. In the present study, BTR was the only BZT with no effect on the molt frequency (Fig 2). The over-transcription of cuticle coding genes could therefore have been the result of a pro-ecdysteroid activity of BTR that acted as a compensation mechanism for the BZT-induced endocrine disruption of molting in Daphnia. In addition, two chitinase and one chitin deacetylase (*cda3*) coding genes were significantly up- and down-regulated in response to BTR, respectively (Fig 4A). Chitin deacetylase is known to influence chitin-protein interactions and chitinases are chitin-degrading enzymes found in the molting fluid and are essential for apolysis and breakdown of the old cuticle and successful completion of the molting cycle [42,89]. Both genes transcriptional response might have influenced alterations in the ultrastructure of the cuticle and could have therefore, along with the over-transcription of cuticle protein coding genes, prevented molting impairment by promoting molting cycle completion.

Two genes coding for a vitellogenin (*vtg*) were among the significantly over-transcribed genes in response to BTR (Fig 4A). VTG is the precursor of the egg-yolk protein vitellin and both proteins accumulates in oocytes during vitellogenesis [90]. Ecdysteroids have been shown to induce vitellogenesis and increase *vtg* mRNA levels in most crustacean species [91,92] and in *D*. *magna*, the down-regulation of *vtg* transcription was observed in response to chronic exposure to JHAs [90] and to perfluoroethylcyclohexane sulfonate [93]. In the latter study, the VTG protein content was also decreased in exposed organisms, along with the up-regulation of cuticle coding genes [93]. The observed *vtg* gene induction in the present study suggests therefore that BTR interferes with endocrine-mediated processes in *D*. *magna*. In addition, two genes coding for sulfotransferases (*sult*) and one for a hydroxysteroid-dehsydrogenase (*hsd*) were also significantly up-regulated by BTR (Fig 4A). SULT and HSD are enzymes involved in steroid hormone biosynthesis in mammals and have been used in fish as biomarkers of endocrine disruption [94], and 3β-HSD has been involved in ecdysteroid biosynthesis in the shore crab [95]. The increase in transcription of both genes in response to BTR could have increased 20HE synthesis and thus explain the up-regulation of 20HE-responding genes such as cuticle proteins and *vtg*.

Among the genes commonly impacted by BTR and 5MeBTR, two chitinases and two cuticular protein coding genes were all up-regulated by BTR and down-regulated by 5MeBTR (S5 Table). This opposite pattern of transcription along with the down-regulation of the majority of molting and 20HE-related genes by 5MeBTR (Fig 4B) clearly indicated distinct and specific effects of both BZTs on endocrine-mediated developmental processes. When over-transcription of cuticle proteins might have prevented molting effects in response to BTR, the present down-regulation of molting genes transcription by 5MeBTR did not support the increased molt frequency observed (Fig 2). These results suggest that different molecular processes not related to cuticle synthesis and metabolism might be responsible for the effects on the number of molts. Among the potential pathways responsible, the gene coding for a histone-lysine N-methyltransferase MLL3 was significantly up-regulated by 5MeBTR exposure (Fig 4B). MLL3 belongs to the histone-modifiers, i.e. a class of epigenetic factors that are involved in drosophila in ecdysone-mediated gene transcription [96]. Epigenetic modifications are known to regulate growth and the formation of helmets and neckteeth in Daphnia, which are exoskeleton extensions used to fend off predators [62]. In addition, a group of 6 homeobox genes were significantly down-regulated in response to 5MeBTR (S5 Table). These genes are highly conserved homeodomain transcription factors involved in essential developmental processes in metazoan, including arthropods [97]. Epigenetic and homeotic processes might therefore represent pathways worth investigating for their role in the increased molt frequency observed following 5MeBTR exposure.

The third BZT, 5ClBTR, affected the lowest number of genes (36 genes; Figs 3 and 4C), but with the highest transcriptional response: three genes coding for molting and 20HE-dependent proteins were differentially transcribed by a factor of 1000 (log_2_FC ± 10; S5 Table). One gene coding for a chitinase was the most up-regulated gene in response to 5ClBTR (Fig 4C). An excessive production of this chitin-degrading enzyme might have altered cuticle production and resulted in the observed decrease in molt frequency (Fig 2). Similar increase in chitinase coding genes was observed in *D*. *magna* after 24-h of genotoxicant exposure [98], and a decrease in chitinase activity was correlated with chronic reproductive effects following exposure to zinc [99]. In addition, one gene coding for an ecdysteroid-regulated protein and for the Kruppel homolog h2 (*kr-h2*) were both down-regulated (Fig 4C). In insects, *Kr-h1* is regulated by JH and represses the transcription of 20HE-induced genes to prevent metamorphosis and maintain the larval status [100]. Although the function of *Kr-h* genes has not yet been studied in crustaceans, the down-regulation of *Kr-h2* in response to 5ClBTR might be the result of JH-mediated perturbations and have contributed to the decreased molting by maintaining the juvenile stage.

Altogether, the transcriptional response of *D*. *magna* to BZTs suggested endocrine-mediated effects on molting and developmental processes.

### Temporal analysis of molting genes transcription

The differentially transcribed genes identified in *D*. *magna* using RNA-seq were further validated by quantitative real-time PCR (qRT-PCR). Specific primers were designed based on the corresponding transcript sequence obtained by RNA-seq for 7 genes that were responding to either one of the three BZTs (S1 Table). The direction of transcription patterns was validated for all selected genes, and the magnitude of differential transcription was confirmed significantly for two genes: *apolipoprotein D* in response to BTR and *Kr-h1* for 5ClBTR (S7 Table).

**Fig 5:**
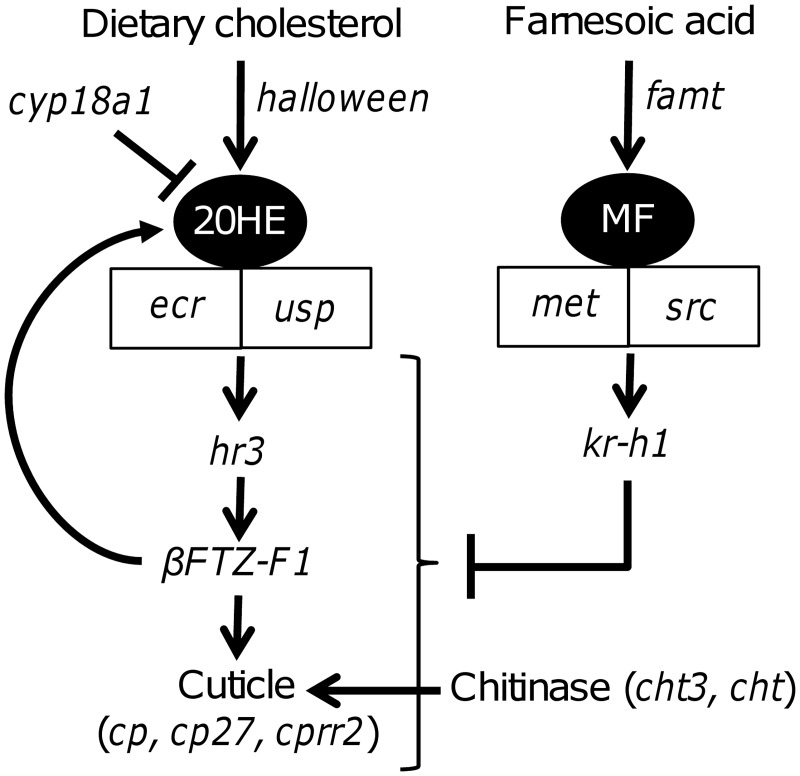
Schematic representation of the major endocrine-mediated pathways involved in molting in *D*. *magna* and some of their associated genes. Acronym definition can be found in S6 Table.

The transcription of a suite of specific candidate genes involved in molting and hormone-dependent processes were further measured by qRT-PCR after 4-, 8- and 21-d of exposure of *D*. *magna* to BZTs in order to identify the molecular pathways involved in molting effects and potential endocrine-disruption. These genes were chosen from a thorough bibliographic search on molting and 20HE-related molecular mechanisms in arthropods with a specific emphasis on Daphnids and crustaceans, and are summarized in Fig 5.

Ecdysteroid and sesquiterpenoid hormones play major roles in the control of molting, growth, development and reproduction in crustaceans [101]. The molting hormone 20HE exerts its action through the binding to the ecdysone receptor (EcR), which heterodimerizes with ultraspiracle (USP) in Daphnia [102] and regulates the transcription of 20HE-responsive genes such as HR3 [75,103,104]. In turn, HR3 is a positive regulator of the transcription factor ßFTZ-F1, which is a major transcriptional activator of cuticle genes in insects [86]. Molting in crustaceans is also regulated to a lesser extent by the sesquiterpenoid hormone methyl farnesoate (MF), the equivalent to insect JH [105], although its precise mode of action is not yet fully understood. In daphnia, recent findings indicate that MF receptor is a heterodimer of two nuclear receptors from the bHLH-PAS family: the methoprene-tolerant receptor (MET) and steroid receptor coactivator (SRC) protein [106]. In insects, these receptors are responsible for the regulation of JH-responsive genes such as *kr-h1* [107,108], which has anti-ecdysteroid activity [100]. However, downstream MF-mediated gene transcription has not yet been investigated in Daphnia and other crustaceans.

**Fig 6:**
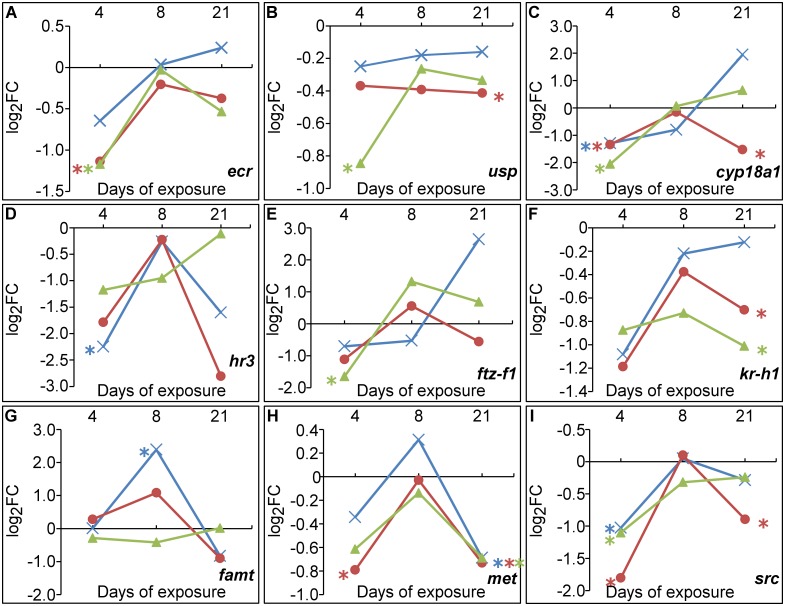
Transcription levels of selected genes related to molting and endocrine-mediated pathways in *D*. *magna* exposed to 2 mg/L of BTR (×), 5MeBTR (●) and 5ClBTR (▲) for 4-, 8- and 21d. (A) *ecr*, (B) *usp*, (C) *cyp18a1*, (D) *hr3*, (E) *ftz-f1*, (F) *kr-h1*, (G) *famt*, (H) *met*, (I) *src*. Gene transcription values are indicated in log_2_FC from qRT-PCR measurements. * indicates a significant difference from the corresponding control (*p*<0.05).

In the present study, 5ClBTR exposure resulted in the down-regulation of *kr-h2* and *kr-h1* genes as measured by RNA-seq and qPCR respectively (Figs 4A and 6F), along with the significant decrease of *met* after 21-d (Fig 6H, S8 Table). In addition, 5ClBTR seemed to induce an increase in the transcription of *cyp18a1* with time, despite a lack of significance (Fig 6C). This enzyme is known to regulate the decline of 20HE titers in Daphnia before molting [109] and could therefore explain the low transcription levels of *ecr* and *usp* observed in the present study (Fig 6A and 6B). These results suggest that the decreased molt frequency observed in response to 5ClBTR seemed to be the result of a perturbation of ecdysteroid signaling pathways rather than MF-mediated anti-ecdysteroid activities. It is worth noting at this point and for the rest of the transcriptomic results that although exposure was initiated with <24-h neonate daphnids, the timing of hormonal regulation of molting is finely tuned and a few hours difference in sampling could have been sufficient to explain the individual variability causing high standard deviations and lack of significance.

On the contrary, 5MeBTR showed a significant down-regulation of *cyp18a1* in response to both doses of exposure (S8 Table) and a down-regulation of *ecr* or *usp* after 4- and 21-d, respectively (Fig 6A, 6B and 6C). In addition, *met*, *src* and *kr-h1* were also significantly down-regulated after 21-d (Fig 6F, 6H and 6I). The increase in molt frequency observed in response to 5MeBTR seemed therefore the result of molecular mechanisms independent of hormonal control. However, as the mode of action of MF and its downstream gene regulation remains to be elucidated, a potential effect of MF cannot be completely ruled out. Complex interactions have been found between MF agonists and the regulation of cuticle proteins with both up- and down-regulation of cuticle protein mRNA levels by MF agonists [87,88,110]. In addition, links between MF and epigenetic and developmental gene transcription as measured by RNA-seq would be worth investigating to explain the present results.

A trend of increase in *cyp18a1* transcription was observed in response to BTR, and was associated with an induction pattern of *ecr* with time (Fig 6A and 6C). These results suggest that the *cyp18a1*-mediated decrease of 20HE levels might have been overcome by other mechanisms, such as the increase of 20HE synthesis induced by *sult* and *hsd* as suggested from the RNA-sequencing results. In addition, the increase of *ftz-f1* transcription was measured over time (Fig 6E). This gene is a transcription factor involved in cuticle gene transcription [86] and in 20HE synthesis [111]. Altogether, these results could confirm the 20HE-increased synthesis as a compensating mechanism in response to BTR. Further measurement of the genes involved in 20HE biosynthesis pathway such as the Halloween gene family [112] could help explain the present results and confirm the increased ecdysteroid synthesis.

### Chitinase activity

**Fig 7:**
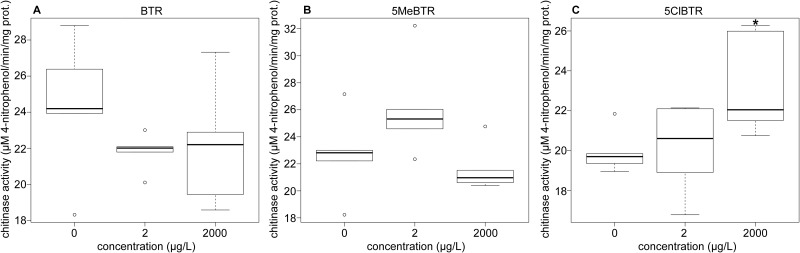
Chitinase activity measured in *D*. *magna* homogenates after 21-d of exposure to 2 and 2000 μg/L of (A) BTR, (B) 5MeBTR and (C) 5ClBTR. * indicates a significant difference from the control (*p*<0.05).

The transcription of genes coding for chitinases were affected by all BZTs, suggesting that they might be good biomarkers of BZT exposure. Two chitinase coding genes, endochitinase-like (*cht*) and chitinase 3 (*cht3*), were both up- and down-regulated by BTR and 5MeBTR respectively (Fig 4A and 4B, S5 Table). Induction patterns were validated by qRT-PCR although not significantly (S7 Table). The gene coding for a brain chitinase and chia (*bcht*), was also highly up-regulated by 5ClBTR (Fig 4C) but this pattern was not reflected by *cht3* transcription measured by qRT-PCR, probably due to the different sequence of the chitinase measured. In order to link the molecular response to the cellular and physiological level, the associated chitinase activity was evaluated in *D*. *magna* exposed to 2 and 2000 μg/L of BTR, 5MeBTR and 5ClBTR for 21-d. Results showed a significant increase in chitinase activity in *D*. *magna* exposed to 2 mg/L of 5ClBTR, thereby confirming the observed differential expression measured by RNA-seq (Fig 7C).

Chitinases are proteolytic enzymes found in molting fluids and responsible for the digestion of the old cuticle during molting, resulting in the successful completion of the molting cycle [89]. Decreases in chitinase mRNA levels and the associated protein activity were measured in *D*. *magna* exposed to metals (Zn and Cu) and further linked to chronic reproductive effects probably due to the cross-talk between molting and reproduction in daphnids [98,113]. In a recent study, chitinase transcription and protein activity were both increased in response to trichloroethylene in *D*. *magna* but no effect on molting frequency was reported [62]. The significant reduction of molt frequency observed in response to 5ClBTR in the present study could be the result of an increased degradation of the cuticle due to the increase in chitinase activity and could represent a potential biomarker of exposure for this chemical. The absence of correlation between molecular and protein responses of chitinases for 5MeBTR and BTR however indicates that post-transcriptional mechanisms might occur and suggest that this enzyme is not linked to the molting effect observed in response to 5MeBTR.

## Conclusion

Chronic exposure of *D*. *magna* to sublethal doses of three BZTs impacted endocrine-mediated processes and molting at the molecular, cellular and physiological level. Each BZT studied showed specific mode of action and endocrine disruption potential, which mostly affected molting. The use of RNA-seq to evaluate the transcriptomic response has proven to be a great tool to investigate the mode of action of BZTs and identify specific molecular pathways that could be linked to the physiological response. The present results have allowed the identification of a suite of biomarker genes associated with endocrine-mediated developmental processes that could be used in future evaluation of toxicity and mode of action of chemicals in Daphnia.

## Supporting information

S1 Protocol Chemical analyses. (DOCX)

S1 Fig Overlap of the genes differentially transcribed (*p*<0.05) in response to 2 mg/L of BTR, 5MeBTR and 5ClBTR following 21-d exposure in *D*. *magna*. (DOCX)

S1 Table Primers used for qRT-PCR experiments in *Daphnia magna*. (DOCX)

S2 Table BZT concentrations measured in spiked culture media. Chemical BZT extraction and analysis were realized between two media renewal.
(DOCX)

S3 Table Summary of sequencing data generated by RNA-seq and *de novo* assembly transcriptome information for *D*. *magna* exposed to BTR, 5MeBTR and 5ClBTR. (DOCX)

S4 Table Assembly statistics of RNA-seq reads. (DOCX)

S5 Table List of all annotated differentially transcribed genes (*p*<0.05) in *Daphnia magna* measured by RNA-sequencing following 21-d individual exposure to 2 mg/L of BTR, 5MeBTR and 5ClBTR. Transcription values are expressed as log_2_ (fold change). Gene over-transcribed are coloured in red and genes under-transcribed in green. N.S.: Non-Significant differential transcription.
(DOCX)

S6 Table List of gene acronyms and their corresponding names from the volcano plot in Fig 4. (DOCX)

S7 Table Gene transcription levels (log_2_FC) of selected genes measured by RNA-seq and qRT-PCR in *D*. *magna* following 21-d exposure to 2 mg/L of BTR, 5MeBTR and 5ClBTR. (DOCX)

S8 Table Gene transcription values (log_2_FC) measured by qRT-PCR in *D*. *magna* exposed for 21-d to 2 and 2000 μg/L of BTR, 5MeBTR and 5ClBTR. Genes were selected based on their differential transcription measured by RNA-seq (genes in bold) and for their role in endocrine-mediated molting processes in Daphnia. Acronym definition can be found in S6 Table.
(DOCX)

## Abbreviations

5ClBTR: 5-chloro-1H-benzotriazole
5MeBTR: 5-methyl-1H-benzotriazole
20HE: 20-hydroxyecdysone
BTR: 1H-benzotriazole
BZTs: benzotriazoles
JH: Juvenile Hormone
JHA: Juvenile Hormone Agonist
MF: Methyl Farnesoate
RNA-seq: RNA-sequencing
