# Supplementary material for: Transcriptomic, cellular and life-history responses of Daphnia magna chronically exposed to benzotriazoles: Endocrine-disrupting potential and molting effects
Source: PLoS One. 2017 Feb 14;12(2):e0171763. doi: 10.1371/journal.pone.0171763 (PMC5308779; doi:10.1371/journal.pone.0171763)
Supplement: S1 Protocol — (DOCX) [file pone.0171763.s001.docx]

**S1 Protocol. Chemical analyses**

Chemical extraction and analysis of BZTs were realized by Environment Canada’s Quebec Laboratory for Environmental Testing (Montreal, Qc, Canada). The quantification was carried out by internal standard method using 2-methylnaphthalene. Results were corrected for recoveries using spike samples at different levels.

*Sample Extraction*

500 mL water samples were spiked with a methanol solution and mixed for 15 minutes. The samples were liquid-liquid extracted three times with 100, 50 and 50 mL of dichloromethane, respectively, for 2 minutes. Sample extracts were then dried on sodium sulfate. The extracts were concentrated to about 2 mL using a rotary evaporator and transferred into 15 mL conical test tubes. The extracts were further reduced to 0.5 mL under a nitrogen stream.

*GC-HRMS determination*

The GC-MS analysis of benzotriazole, 5-methylbenzotriazole and 5-chlorobenzotriazole was carried out on a 7690 GC (Agilent) coupled to a AutoSpec Premier (Waters). The chromatographic separation was performed on a 60 meters DB5 column (J&W Scientific), 0.25 mm I.D., 0.25 µm phase thichness, fitted with an uncoated fused-silica guard column of 2 m X 0.53 mm I.D. (Restek). The oven temperature was programmed from 100 ^o^C (1 minute) to 210 ^o^C at a rate of 30 ^o^C/min (8 minutes hold time). Helium was used as the carrier gas at 1.5 mL/min. Samples were injected in a Cool-OnColumn injector held at 100 ^o^C for 1 minute then ramped to 320 ^o^C at 100 ^o^C/min. The MS was operated in the electron ionization mode (EI) at 35 eV electron energy. Transfer line and source temperature were set at 275 and 200 ^o^C, respectively. Data were acquired at 10 000 resolution (5% peak height). The quantification was carried out by internal standard method using 2-methylnaphthalene. Results were corrected for recoveries using spike samples at different levels.
