# Supplementary material for: Transcriptomic, cellular and life-history responses of Daphnia magna chronically exposed to benzotriazoles: Endocrine-disrupting potential and molting effects
Source: PLoS One. 2017 Feb 14;12(2):e0171763. doi: 10.1371/journal.pone.0171763 (PMC5308779; doi:10.1371/journal.pone.0171763)

**S1 Fig. Overlap of the genes differentially transcribed (*p*<0.05) in response to 2 mg/L of BTR, 5MeBTR and 5ClBTR following 21-d exposure in *D. magna*.**


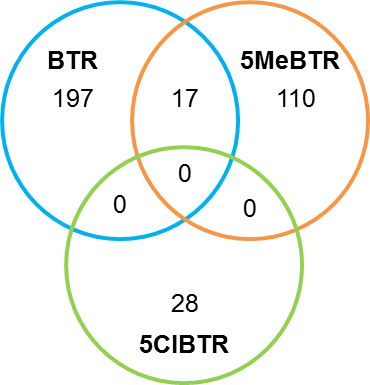

Supplement: S1 Fig — (DOCX) [file pone.0171763.s002.docx]
