# Supplementary material for: Transcriptomic, cellular and life-history responses of Daphnia magna chronically exposed to benzotriazoles: Endocrine-disrupting potential and molting effects
Source: PLoS One. 2017 Feb 14;12(2):e0171763. doi: 10.1371/journal.pone.0171763 (PMC5308779; doi:10.1371/journal.pone.0171763)
Supplement: S1 Table — (DOCX) [file pone.0171763.s003.docx]

**S1 Table. Primers used for qRT-PCR experiments in *Daphnia magna*.**

| Gene name | symbol | Primer sequence 5’-3’ | Amplicon length (bp) | Efficiency (%) | Reference |
| --- | --- | --- | --- | --- | --- |
| **RNA-seq validation** |  |  |  |  |  |
| Cuticular protein RR-2 motif 132 | *cprr2* | F-ACCACCAGCAAACCTTACAC | 135 | 96 | this study |
|  |  | R-CCATCCGTTGTCATCTCCTT |  |  |  |
| Cuticular protein | *cp* | F-TCTGCTGGGAAAGTTGAAGATG  R-CAAATGGACGAGAAGACGATG | 79 | 94 | this study |
| Cuticular protein 27 | *cp27* | F-CCTGTCCGTCCCGTCTTTCT  R-GGTAGTGGTGCTGGGATGGA | 136 | 93 | this study |
| T-complex protein 1 subunit delta | *cct4* | F-TCAGTCAGGTAAAGGAGATGTG  R-GCCAGCAAGAACCACAACA | 160 | 94 | this study |
| Apolipoprotein D | *apoD* | F-CCGTTTCTTGGAGTTGTATTGG  TTTCGGGGGTTGGATTTGGA | 87 | 98 | this study |
| Endochitinase-like | *cht* | F-CAGAGCAAATAGGGCGAGAG  R- GCGATTGTGCCGTGTATTT | 82 | 97 | this study |
| Chitinase 3 | *cht3* | F- TGTTGCTTTTTGATCGCGCA | 68 | 109 | this study |
|  |  | R- AATGCAGGAGGAAGTGTCGG |  |  |  |
| **20E-dependent and molting genes** | |  |  |  |  |
| Ecdysone receptor | *ecr* | F- GAGGCGCTGCAGGCTTAC | 63 | 102 | [[1](#_ENREF_1)] |
|  |  | R- GAGTTTGGCAAACTCCGTCATC |  |  |  |
| Ultraspiracle | *usp* | F-GTTGGAGTCAAGGATGGTATCGT | 60 | 103 | [[1](#_ENREF_1)] |
|  |  | R- AGCCGAGTTCCGGTGGAT |  |  |  |
| Cytochrome 18A1 | *cyp18a1* | F- TACCCGATCGTCGGTTACCT | 63 | 94 | [[1](#_ENREF_1)] |
|  |  | R- GAGCGCCGTCAGCTCTTC |  |  |  |
| Nuclear hormone receptor HR3 | *hr3* | F- AAGGTCGAGGATGAAGTGCG | 81 | 95 | [[2](#_ENREF_2)] |
|  |  | R- AAAGACGCTACTATCGGGCG |  |  |  |
| Nuclear hormone receptor FTZ-F1 | *ftz-f1* | F- TCTTACCGGACATTCACGCC | 71 | 105 | [[3](#_ENREF_3)] |
|  |  | R- ACAGCCGTTGAGATGCTTGA |  |  |  |
| Krueppel homolog 1 | *kr-h1* | F- TCATCTTGGCGAGCGATTGT | 75 | 93 | this study |
|  |  | R- TGAGCCTCCAGCGTTTTCTT |  |  |  |
| Farnesoic acid carboxyl-O-methyltransferase | *famt* | F- TTACCAGTACGCTGCTCGAC | 69 | 98 | this study |
|  |  | R- ACCACCTGCAAACCAACTGA |  |  |  |
| Methoprene tolerant receptor | *met* | F- CAAACAGCCAGAGATTACCGG | 81 | 98 | [[2](#_ENREF_2)] |
|  |  | R- GCACTGTTGGTTCCAGCATTC |  |  |  |
| Steroid receptor coactivator | *src* | F- TACTAGGCGTCTTGCTGAATGAA | 81 | 104 | [[2](#_ENREF_2)] |
|  |  | R- CCATAATTTGCAAGGCTCCG |  |  |  |
| **Reference genes** | |  |  |  |  |
| Tata box binding protein | *tata* | F-ACCCGAAAGCACAAATCAGCGA | 178 | 97 | this study |
|  |  | R-GCGCCAAAGTTCGTCAAGAGA |  |  |  |
| Glyceraldehyde-3-phosphate dehydrogenase | *gapdh* | F- TGCTGATGCCCCAATGTTTGTTGT | 132 | 92 | this study |
|  |  | R-GCAGTTATGGCGTGGACGGTTGT |  |  |  |
| Ubiquitin | *ub* | F- ACCACACGCATCTATCATCCCAA | 174 | 91 | this study |
|  |  | R- TGGGTCGCATAGCAGAGAACA |  |  |  |
