# Supplementary material for: Transcriptomic, cellular and life-history responses of Daphnia magna chronically exposed to benzotriazoles: Endocrine-disrupting potential and molting effects
Source: PLoS One. 2017 Feb 14;12(2):e0171763. doi: 10.1371/journal.pone.0171763 (PMC5308779; doi:10.1371/journal.pone.0171763)
Supplement: S2 Table — Chemical BZT extraction and analysis were realized between two media renewal. (DOCX) [file pone.0171763.s004.docx]

**S2 Table.** **BZT concentrations measured in spiked culture media.** Chemical BZT extraction and analysis were realized between two media renewal.

|  |  | Measured concentration (µg/L) | Expected concentration (µg/L) | Measured concentration (µg/L) | Expected concentration (µg/L) |
| --- | --- | --- | --- | --- | --- |
| BTR | Day 0 | 0.75 | 2 | 2700 | 2000 |
|  | Day 2 | 2.2 | 2 | 2400 | 2000 |
| 5MeBTR | Day 0 | 2.2 | 2 | 1900 | 2000 |
|  | Day 4 | 2.8 | 2 | 1800 | 2000 |
| 5ClBTR | Day 0 | 2.8 | 2 | 2900 | 2000 |
|  | Day 3 | 3 | 2 | 2800 | 2000 |
