# Supplementary material for: Transcriptomic, cellular and life-history responses of Daphnia magna chronically exposed to benzotriazoles: Endocrine-disrupting potential and molting effects
Source: PLoS One. 2017 Feb 14;12(2):e0171763. doi: 10.1371/journal.pone.0171763 (PMC5308779; doi:10.1371/journal.pone.0171763)
Supplement: S3 Table — (DOCX) [file pone.0171763.s005.docx]

**S3 Table.** **Summary of sequencing data generated by RNA-seq and *de novo* assembly transcriptome information for *D. magna* exposed to BTR, 5MeBTR and 5ClBTR.**

| **Compound** | **Treatment** | **Raw nucleotides** | **Raw paired reads** | **Clean paired reads** |
| --- | --- | --- | --- | --- |
| **BTR** | **control** | 22,527,006,600 | 112,635,033 | 106,502,460 |
|  | **2 mg/L** | 23,651,426,000 | 118,257,130 | 111,491,965 |
| **5MeBTR** | **control** | 22,998,283,000 | 114,991,415 | 108,843,496 |
|  | **2 mg/L** | 22,753,826,000 | 113,769,130 | 107,469,528 |
| **5ClBTR** | **control** | 20,722,934,400 | 103,614,672 | 99,125,804 |
|  | **2 mg/L** | 20,146,566,200 | 100,732,831 | 95,963,860 |
| **Total** |  | 132,800,042,200 | 664,000,211 | 629,397,113 |
