# Supplementary material for: Transcriptomic, cellular and life-history responses of Daphnia magna chronically exposed to benzotriazoles: Endocrine-disrupting potential and molting effects
Source: PLoS One. 2017 Feb 14;12(2):e0171763. doi: 10.1371/journal.pone.0171763 (PMC5308779; doi:10.1371/journal.pone.0171763)
Supplement: S4 Table — (DOCX) [file pone.0171763.s006.docx]

**S4 Table. Assembly statistics of RNA-seq reads.**

| **Parameter** | **Value** |
| --- | --- |
| **Number of transcripts** | 41,538 |
| **Number of components** | 14,666 |
| **Minimum transcript length (bp)** | 224 |
| **Mean transcript length (bp)** | 2385 |
| **Maximum transcript length (bp)** | 23,779 |
| **N50 (bp)** | 3,263 |
| **%GC** | 44 |
