# Supplementary material for: Transcriptomic, cellular and life-history responses of Daphnia magna chronically exposed to benzotriazoles: Endocrine-disrupting potential and molting effects
Source: PLoS One. 2017 Feb 14;12(2):e0171763. doi: 10.1371/journal.pone.0171763 (PMC5308779; doi:10.1371/journal.pone.0171763)
Supplement: S5 Table — Transcription values are expressed as log2 (fold change). Gene over-transcribed are coloured in red and genes under-transcribed in green. N.S.: Non-Significant differential transcription. (DOCX) [file pone.0171763.s007.docx]

**S5 Table. List of all annotated differentially transcribed genes (*p*<0.05) in *Daphnia magna* measured by RNA-sequencing following 21-d individual exposure to 2 mg/L of BTR, 5MeBTR and 5ClBTR.** Transcription values are expressed as log_2_ (fold change). Gene over-transcribed are coloured in red and genes under-transcribed in green. N.S.: Non-Significant differential transcription.

| **Transcript ID** | | **Predicted function** | |  |  | **Log_2_ FC ([BZT] vs [Control])** | | | | |
| --- | --- | --- | --- | --- | --- | --- | --- | --- | --- | --- |
|  |  |  |  |  |  | **BTR** | | **5MeBTR** | **5ClBTR** | |
| **Molting** | |  | | | |  |  |  | | |
| TR2454\|c0_g1_i4 | | Chitin deacetylase 3 precursor | | | | -2,462 | NS | NS | | |
| TR12386\|c1_g2_i1 | | Cuticular protein | | | | -2,264 | NS | NS | | |
| TR17193\|c0_g1_i1 | | Cuticular protein analogous to peritrophins 1-G | | | | -2,265 | NS | NS | | |
| TR18327\|c0_g1_i1 | | Cuticlin-1 | | | | -2,007 |  |  | | |
| TR3539\|c0_g1_i2 | | Cuticular protein analogous to peritrophins 1-D | | | | 2,415 |  |  | | |
| TR18059\|c0_g1_i1 | | Sulfotransferase sult | | | | 2,548 | NS | NS | | |
| TR14873\|c0_g1_i1 | | Sulfotransferase family cytosolic 1B member | | | | 2,408 | NS | NS | | |
| TR5495\|c0_g1_i4 | | Cuticular protein 72Eb | | | | 3,589 | NS | NS | | |
| TR5495\|c0_g2_i1 | | Cuticular protein 72Eb | | | | 5,054 | NS | NS | | |
| TR16595\|c1_g1_i1 | | Cuticular protein | | | | 3,443 | NS | NS | | |
| TR7965\|c0_g2_i1 | | Cuticle protein 16.5, isoform B-like | | | | 3,358 | NS | NS | | |
| TR18670\|c0_g1_i2 | | Cuticle protein 7 | | | | 3,898 | NS | NS | | |
| TR21222\|c0_g1_i1 | | Cuticular protein | | | | 3,948 | NS | NS | | |
| TR5360\|c0_g1_i1 | | Cuticle protein | | | | 7,907 | -2,53 | NS | | |
| TR315\|c0_g1_i2 | | Endochitinase-like | | | | 5,244 | -2,625 | NS | | |
| TR10295\|c0_g1_i1 | | Cuticular protein 27 | | | | 5,649 | NS | NS | | |
| TR1915\|c0_g1_i1 | | Cuticle protein | | | | 7,044 | NS | NS | | |
| TR21858\|c0_g1_i6 | | Chitinase 3 | | | | 7,078 | -3,326 | NS | | |
| TR26908\|c0_g1_i2 | | Cuticular protein RR-2 motif 132 | | | | 10,495 | -2,25 | NS | | |
| TR27488\|c0_g1_i1 | | Cuticle protein | | | | NS | -2,339 | NS | | |
| TR10948\|c0_g1_i1 | | Cuticle protein | | | | NS | -2,293 | NS | | |
| TR5693\|c0_g1_i1 | | cuticular protein | | | |  | -2,004 |  | | |
| TR15668\|c0_g2_i1 | | Brain chitinase and chia | | | | NS | NS | 10,468 | | |
| **Glycan biosynthesis and metabolism** | | | | | |  |  |  | | |
| TR17500\|c0_g1_i1 | | Alpha-(1,3)-fucosyltransferase | | | | 5,199 | NS | NS | | |
| TR9540\|c0_g1_i1 | | Alpha-(1,3)-fucosyltransferase 11 | | | | -2,966 | NS | NS | | |
| TR4804\|c0_g1_i1 | | Beta-galactoside alpha-2,6-sialyltransferase | | | | -2,27 | NS | NS | | |
| TR21409\|c0_g1_i1 | | Beta-1,4-galactosyltransferase | | | | NS | -3,493 | NS | | |
| TR27685\|c1_g2_i1 | | Lactosylceramide 4-alpha-galactosyltransferase | | | | NS | -2,828 | NS | | |
| TR2230\|c0_g1_i2 | | Lactosylceramide | | | | NS | -2,431 | NS | | |
| TR5282\|c0_g1_i1 | | Beta-1,3-galactosyltransferase 1 | | | | NS | NS | -5,958 | | |
| **20E-related mechanisms** | | | | | |  |  |  | | |
| TR7253\|c0_g1_i1 | | Hydroxysteroid dehydrogenase | | | | 2,3 | NS | NS | | |
| TR24696\|c0_g1_i1 | | Vitellogenin- fused with superoxide dismutase | | | | 3,534 | NS | NS | | |
| TR25602\|c0_g2_i2 | | Vitellogenin-1 precursor | | | | 2,791 | NS | NS | | |
| TR18077\|c1_g1_i1 | | Vitellogenin-1 like | | | |  |  | -10,325 | | |
| TR25192\|c1_g2_i1 | | ATP-dependent RNA helicase DDX4 | | | | 4,787 | NS | NS | | |
| TR870\|c0_g1_i1 | | Histone-lysine N-methyltransferase SETD2 | | | | 3,28 | NS | NS | | |
| TR28161\|c1_g1_i1 | | Histone-lysine N-methyltransferase SETD2 | | | | 4,691 | NS | NS | | |
| TR2078\|c1_g2_i1 | | Histone-lysine N-methyltransferase MLL3 | | | | NS | 3,413 | NS | | |
| TR18241\|c0_g1_i1 | | Histone-lysine N-methyltransferase NSD2-like protein | | | |  | -4,041 |  | | |
| TR21048\|c0_g2_i1 | | Receptor-type tyrosine-protein phosphatase alpha precursor | | | | NS | -5,714 | NS | | |
| TR25581\|c0_g1_i1 | | ecdysteroid-regulated 16 kDa protein (NPC2) | | | |  |  | -10,865 | | |
| TR1754\|c0_g1_i1 | | Kr-h2 (Krueppel homolog) | | | | NS | NS | -7,356 | | |
| **Structural proteins** | | | | | |  |  |  | | |
| TR9222\|c0_g1_i2 | | Collagen alpha-1(IV) chain | | | | -2,68 | NS | NS | | |
| TR7499\|c1_g1_i1 | | Contactin associated protein 1 | | | | -2,356 | NS | NS | | |
| TR7499\|c2_g3_i2 | | Contactin associated protein 1 | | | | -3,641 | NS | NS | | |
| TR18327\|c0_g1_i1 | | Cuticlin-1 | | | | -2,007 | NS | NS | | |
| TR18739\|c0_g1_i1 | | Collagen alpha-1(XXIII) chain | | | | NS | -2,841 | NS | | |
| **Development / cell morphogenesis** | | | | | | | | | | |
| TR9106\|c2_g2_i1 | | Homeobox protein cut | | | | -2,134 | NS | NS | | |
| TR6299\|c0_g1_i1 | | Membrane glycoprotein lig-1 | | | | -2,133 | NS | NS | | |
| TR12987\|c0_g2_i1 | | Twisted gastrulation protein-A | | | | -5,945 | NS | NS | | |
| TR15724\|c0_g1_i1 | | Germinal histone H4 protein | | | | 2,91 | NS | NS | | |
| TR5622\|c2_g3_i1 | | Homeotic HOX3 protein | | | | 3,284 | -5,344 | NS | | |
| TR5710\|c2_g1_i1 | | Nuclear distribution protein nudE 1-B | | | | 4,692 | NS | NS | | |
| TR3040\|c0_g1_i1 | | Secreted frizzled-related protein | | | | 4,782 | NS | NS | | |
| TR6370\|c0_g1_i1 | | Nose resistant to fluoxetine protein | | | | 4,981 | -2,582 | NS | | |
| TR25522\|c0_g1_i1 | | Innexin | | | | NS | -4,666 | NS | | |
| TR27724\|c1_g2_i1 | | Homeobox even-skipped protein | | | | NS | -3,756 | NS | | |
| TR16518\|c0_g1_i1 | | Homeobox protein Hox-A7 | | | | NS | -3,487 | NS | | |
| TR7976\|c0_g1_i1 | | T-box transcription factor TBX2 | | | | NS | -2,815 | NS | | |
| TR5312\|c0_g1_i1 | | Homeobox protein goosecoid | | | | NS | -2,528 | NS | | |
| TR15398\|c1_g1_i2 | | Homeobox protein orthopedia B | | | | NS | -2,415 | NS | | |
| TR20697\|c3_g2_i1 | | H3 histone | | | | NS | -2,375 | NS | | |
| TR1015\|c1_g1_i1 | | Homeobox protein Hox-C4 | | | | NS | -2,198 | NS | | |
| TR18336\|c3_g1_i2 | | Histone h2a | | | | NS | -2,198 | NS | | |
| TR14619\|c0_g1_i1 | | Bone morphogenetic protein | | | | NS | -2,091 | NS | | |
| TR12052\|c0_g2_i1 | | Uncharacterized protein | | | | NS | 4,93 | NS | | |
| TR21224\|c0_g2_i1 | | DNA topoisomerase I | | | | NS | 8,471 | NS | | |
| TR27896\|c1_g1_i1 | | Heat shock 70 kDa protein cognate 4 | | | | NS | NS | -10,06 | | |
| **Lipid metabolism** | |  | | | |  |  |  | | |
| TR21790\|c0_g1_i1 | | Apolipoprotein D | | | | -8,436 | NS | NS | | |
| TR11768\|c0_g1_i1 | | Apolipoprotein D | | | | -4,744 | NS | NS | | |
| TR1240\|c0_g1_i1 | | Apolipoprotein D | | | | -3,203 | NS | NS | | |
| TR9148\|c1_g1_i1 | | Apolipoprotein D | | | | -2,951 | NS | NS | | |
| TR28166\|c3_g2_i1 | | Lipase 3 | | | | -4,44 | NS | NS | | |
| TR28166\|c1_g1_i1 | | Lipase 3 | | | | -2,207 | NS | NS | | |
| TR14846\|c4_g1_i1 | | SEC14-like protein | | | | -2,699 | NS | NS | | |
| TR27517\|c4_g1_i1 | | 4-coumarate--CoA ligase | | | | 5,015 | NS | NS | | |
| TR13860\|c0_g1_i1 | | Secretory phospholipase a2 | | | | 6,068 | -2,473 | NS | | |
| TR6240\|c0_g1_i2 | | Lipase | | | | NS | -2,097 | NS | | |
| TR7303\|c0_g1_i1 | | Pancreatic triacylglycerol lipase | | | | NS | 4,972 | NS | | |
| TR27416\|c0_g2_i1 | | Phospholipase A-2-activating protein | | | | NS | NS | -4,685 | | |
| TR6718\|c0_g1_i2 | | C1q-like adipose specific protein-like protein | | | | NS | NS | -2,266 | | |
| TR28100\|c0_g1_i1 | | Elongation of very long chain fatty acids protein | | | | NS | NS | 5,282 | | |
| **Protein metabolism** | | | | | | | | | | |
| TR27710\|c0_g1_i1 | | Zinc carboxypeptidase | | | | -4,838 | NS | NS | | |
| TR9749\|c0_g1_i1 | | ATP-dependent zinc metalloprotease | | | | -4,585 | NS | NS | | |
| TR13671\|c0_g1_i1 | | E3 ubiquitin-protein ligase rififylin | | | | -3,432 | NS | NS | | |
| TR8030\|c0_g1_i1 | | Serine protease inhibitor | | | | -2,185 | NS | NS | | |
| TR5959\|c1_g1_i6 | | Serine threonine-protein kinase | | | | 2,061 | NS | NS | | |
| TR5959\|c3_g3_i2 | | Serine threonine-protein kinase | | | | 2,476 | NS | NS | | |
| TR20749\|c0_g1_i2 | | Serine threonine-protein kinase | | | | 2,504 | NS | NS | | |
| TR5959\|c3_g2_i1 | | Serine threonine-protein kinase | | | | 2,632 | NS | NS | | |
| TR19361\|c0_g1_i1 | | Clip-domain serine protease | | | | 2,721 | NS | NS | | |
| TR21324\|c0_g1_i1 | | Proline dehydrogenase 1, mitochondrial | | | | 4,681 | NS | NS | | |
| TR20230\|c1_g2_i1 | | Ubiquitin modifier-activating enzyme | | | | 5,363 | NS | NS | | |
| TR11178\|c0_g1_i1 | | Ubiquitin protein 7 | | | | 5,613 | NS | NS | | |
| TR11056\|c0_g2_i1 | | 26S proteasome non-ATPase regulatory subunit 8 | | | | NS | -5,18 | NS | | |
| TR18909\|c0_g1_i1 | | Serine/threonine-protein kinase | | | | NS | -4,83 | NS | | |
| TR9794\|c0_g1_i1 | | Tryptophan 5-hydroxylase | | | | NS | -2,928 | NS | | |
| TR1803\|c2_g8_i15 | | Ubiquitin-conjugating enzyme E2 | | | | NS | -2,418 | NS | | |
| TR14948\|c1_g2_i1 | | Ubiquitin conjugating enzyme 7 | | | | NS | 5,105 | NS | | |
| TR19738\|c0_g3_i2 | | Cathepsin L | | | | NS | NS | -4,074 | | |
| TR3525\|c0_g2_i1 | | Trypsin 1-like | | | | NS | NS | -2,039 | | |
| **Energy metabolism** | | | | | | | | | | |
| TR4219\|c0_g1_i1 | | Solute carrier family 2, facilitated glucose | | | | -4,522 | NS | NS | | |
| TR12179\|c1_g2_i1 | | Glucose dehydrogenase [FAD, quinone] | | | | -3,189 | NS | NS | | |
| TR23459\|c0_g1_i1 | | Neutral alpha-glucosidase C | | | | 7,516 | NS | NS | | |
| TR24447\|c0_g1_i1 | | Fructose-bisphosphate aldolase isoform X2 | | | |  | NS | -9,802 | | |
| **RNA processing and metabolism** | | | | | | | | | | |
| TR14005\|c0_g1_i1 | | Tudor and KH domain-containing protein | | | | -2,117 | NS | NS | | |
| TR18205\|c0_g2_i1 | | RNA pseudouridylate synthase | | | | 5,484 | NS | NS | | |
| TR5591\|c0_g1_i1 | | THO complex subunit | | | | 11,924 | NS | NS | | |
| TR18209\|c0_g2_i1 | | RNase_HI_RT_Ty3, Ty3/Gypsy family of RNase HI in long-term repeat | | | | 2,547 |  | |  | |
| **Transcription / Translation** | | | | | | | | | | |
| TR23915\|c0_g1_i1 | | Translation initiation factor IF-2 | | | | -4,925 | NS | NS | | |
| TR10999\|c0_g1_i1 | | 28S ribosomal protein S30, mitochondrial | | | | -2,083 | NS | NS | | |
| TR19840\|c0_g1_i1 | | Spz1 | | | | -2,571 | NS | NS | | |
| TR23039\|c4_g1_i1 | | Transcription factor Sox-14 | | | | -2,268 | NS | NS | | |
| TR25096\|c0_g2_i1 | | Transcription factor atf1 | | | | 6,482 | -2,233 | NS | | |
| TR9119\|c0_g1_i1 | | Basic-leucine zipper transcription regulator giant | | | | 7,765 | -3,715 | NS | | |
| TR11306\|c0_g1_i1 | | Zinc finger transcription factor | | | | NS | -3,183 | NS | | |
| TR23018\|c1_g1_i1 | | Huckebein | | | | NS | -3,137 | NS | | |
| TR8012\|c0_g2_i1 | | Reverse transcriptase | | | | NS | 5,998 | NS | | |
| TR10734\|c0_g2_i1 | | Eukaryotic translation initiation factor 3 subunit C | | | | NS | NS | -5,953 | | |
| TR23377\|c0_g2_i1 | | tRNA pseudouridine synthase 1 | | | | NS | NS | -2,111 | | |
| **Cytoskeleton** | | | | | | | | | | |
| TR9879\|c1_g1_i1 | | Myosin-2 heavy chain | | | | 2,161 | NS | NS | | |
| TR12861\|c0_g1_i1 | | Actin-related protein | | | | 6,444 | NS | NS | | |
| TR6948\|c0_g2_i1 | | T-complex protein 1 subunit delta | | | | NS | -9,555 | NS | | |
| TR7628\|c0_g2_i1 | | T-complex protein 1 subunit beta | | | | NS | -7,188 | NS | | |
| TR9360\|c0_g1_i1 | | Unconventional myosin-X | | | | NS | -2,867 | NS | | |
| **Oxidative stress** | | | | | | | | | | |
| TR9613\|c0_g1_i1 | | Peroxidase chorion | | | | -2,454 | NS | NS | | |
| TR23555\|c0_g1_i1 | | Guanine deaminase | | | | 4,498 | NS | NS | | |
| TR26585\|c0_g1_i1 | | Peroxidase precursor | | | | 6,454 | NS | NS | | |
| TR28979\|c0_g1_i1 | | Hexaprenyldihydroxybenzoate methyltransferase | | | | NS | NS | 3,784 | | |
| **Ion transport, homeostasis** | | | | | | | | | | |
| TR15362\|c0_g1_i1 | | GTP-binding protein CG1354 | | | | 2,446 | NS | NS | | |
| TR4611\|c0_g1_i21 | | Solute carrier family 13 member | | | | 2,652 | NS | NS | | |
| TR131\|c0_g1_i5 | | Glutamate receptor, ionotropic kainate 2 | | | | 2,727 | NS | NS | | |
| TR21608\|c0_g1_i1 | | Zinc/iron regulated transporter-related protein | | | | 2,75 | NS | NS | | |
| TR17516\|c0_g2_i2 | | Voltage-dependent T-type calcium channel subunit alpha-1G | | | | 3,679 | NS | NS | | |
| TR23527\|c0_g2_i1 | | V-type proton ATPase subunit C 1-A | | | | 6,226 | 2,285 | NS | | |
| TR25665\|c1_g4_i1 | | Cation-transporting atpase | | | | NS | 6,083 | NS | | |
| TR1472\|c1_g1_i2 | | Na(+)/Pi cotransporter | | | | NS | -6,73 | NS | | |
| **Membrane trafficking** | | | | | | | | | | |
| TR7572\|c0_g1_i1 | | Myotubularin-related protein | | | | NS | -7,115 | NS | | |
| TR13755\|c0_g2_i1 | | Trafficking protein particle complex subunit 6B | | | | NS | NS | -2,186 | | |
| **Response to drug** | | | | | | | | | | |
| TR25447\|c4_g1_i1 | | ATP-binding cassette sub-family A | | | | NS | -3,308 | NS | | |
| TR16607\|c0_g1_i1 | | Glucosyl/glucuronosyl transferases | | | | NS | -2,834 | NS | | |
| **Retinol metabolism** | | | | | | | | | | |
| TR4143\|c0_g1_i1 | | Retinol dehydrogenase | | | | NS | -2,439 | NS | | |
| **Immune response** | | | | | | | | | | |
| TR25023\|c0_g2_i1 | | Complement C1q tumor necrosis factor-related protein 3 NS | | | | | -2,997 | NS | | |
| TR20856\|c0_g1_i1 | | C-type lectin ctl - mannose binding | | | | NS | 2,116 | NS | | |
| **Other functions** | | | | | | | | | | |
| TR24174\|c0_g1_i1 | | Tetraspanin-11 | | | | -2,985 | NS | NS | | |
| TR15017\|c0_g2_i1 | | LisH domain-containing protein FOPNL | | | | -4,836 | NS | NS | | |
| TR14809\|c0_g1_i3 | | Heme-binding protein 2 | | | | -2,238 | NS | NS | | |
| TR13056\|c1_g1_i2 | | Zinc finger CCCH domain-containing protein 4-like | | | | -2,263 | NS | NS | | |
| TR11453\|c0_g1_i1 | | DmX protein 2 | | | | -5,847 | NS | NS | | |
| TR16609\|c0_g3_i1 | | Dexamethasone-induced Ras-related | | | | -2,366 | NS | NS | | |
| TR17987\|c0_g2_i1 | | Methyltransferase-like protein 13 | | | | 2,371 | NS | NS | | |
| TR14563\|c0_g1_i3 | | Carbonic anhydrase | | | | 2,424 | NS | NS | | |
| TR14862\|c0_g1_i1 | | SecG, Translocase, SecG subunit | | | | 2,514 | NS | NS | | |
| TR19222\|c0_g2_i2 | | Denn domain-containing protein | | | | 2,773 | NS | NS | | |
| TR26247\|c0_g1_i1 | | Reverse transcriptase from retrotransposon | | | | 3,642 | NS | NS | | |
| TR7838\|c0_g1_i2 | | Lethal2essential for life protein | | | | 4,034 | NS | NS | | |
| TR3086\|c1_g2_i1 | | DENN domain-containing protein 5B-like | | | | 4,607 | NS | NS | | |
| TR14174\|c0_g2_i1 | | Nuclear pore complex protein Nup93 | | | | 6,584 | NS | NS | | |
| TR19541\|c0_g1_i1 | | Prolactin regulatory element-binding protein | | | | 7,215 | NS | NS | | |
| TR9874\|c0_g1_i1 | | Gmp synthase | | | | 2,181 | NS | NS | | |
| TR11132\|c0_g1_i1 | | Dickkopf-related protein | | | | 7,826 | NS | NS | | |
| TR2305\|c0_g6_i1 | | WD repeat-containing protein | | | | NS | -6,592 | NS | | |
| TR17210\|c0_g2_i1 | | N-acetyltransferase | | | | NS | -6,077 | NS | | |
| TR5724\|c0_g3_i1 | | Integrase | | | | NS | -5,005 | NS | | |
| TR2642\|c0_g2_i1 | | Aldehyde oxidase | | | | NS | -4,949 | NS | | |
| TR18585\|c0_g2_i1 | | Cytochrome b5 | | | | NS | -4,745 | NS | | |
| TR16837\|c0_g2_i1 | | 5'-nucleotidase domain-containing protein | | | | NS | -3,02 | NS | | |
| TR7886\|c0_g2_i1 | | CLK4-associating serine/arginine | | | | NS | -2,104 | NS | | |
| TR21447\|c0_g1_i1 | | ZZ-type zinc finger-containing protein | | | | NS | -3,787 | NS | | |
| TR14726\|c0_g1_i1 | | TBC1 domain family member | | | | NS | -2,759 | NS | | |
| TR28130\|c1_g1_i1 | | Dystrophin | | | | NS | 2,072 | NS | | |
| TR22904\|c0_g2_i1 | | Transposase | | | | NS | 2,673 | NS | | |
| TR13893\|c0_g1_i1 | | Mismatch repair endonuclease PMS2 | | | | NS | 5,077 | NS | | |
| TR21015\|c0_g2_i1 | | Class a rhodopsin g-protein coupled receptor gprnna7 | | | | NS | 5,11 | NS | | |
