# Supplementary material for: Transcriptomic, cellular and life-history responses of Daphnia magna chronically exposed to benzotriazoles: Endocrine-disrupting potential and molting effects
Source: PLoS One. 2017 Feb 14;12(2):e0171763. doi: 10.1371/journal.pone.0171763 (PMC5308779; doi:10.1371/journal.pone.0171763)
Supplement: S6 Table — (DOCX) [file pone.0171763.s008.docx]

**S6 Table. List of gene acronyms and their corresponding names from the volcano plot in Fig 4.**

| **Gene acronym** | **Gene name** |
| --- | --- |
| *cda3* | Chitin deacetylase 3 |
| *cht* | Endochitinase-like |
| *cht3* | Chitinase 3 |
| *cp* | Cuticle protein |
| *cp27* | Cuticle protein 27 |
| *cp72eb* | Cuticular protein 72Eb |
| *cp7* | Cuticular protein 7 |
| *cpap* | Cuticle protein analog to peritrophin |
| *cpb* | Cuticle protein 16.5, isoform B-like |
| *cprr2* | Cuticular protein RR-2 motif 132 |
| *cut-1* | Cuticlin-1 |
| *ddx4* | ATP-dependent RNA helicase DDX4 |
| *hsd* | Hydroxysteroid dehydrogenase |
| *kr-h2* | Kruppel-homolog 2 |
| *mll3* | Histone-lysine N-methyltransferase MLL3 |
| *npc2* | Ecdysteroid-regulated 16 kDa protein (NPC2) |
| *nsd2* | Histone-lysine N-methyltransferase NSD2-like protein |
| *ptpra* | Receptor-type tyrosine-protein phosphatase alpha precursor |
| *setd2* | Histone-lysine N-methyltransferase SETD2 |
| *sult* | Sulfotransferase |
| *sult1b* | Sulfotransferase family cytosolic 1B member |
| *vtg1* | Vitellogenin-1 |
| *whc1* | Histone-lysine N-methyltransferase NSD2-like protein |
