# Supplementary material for: Transcriptomic, cellular and life-history responses of Daphnia magna chronically exposed to benzotriazoles: Endocrine-disrupting potential and molting effects
Source: PLoS One. 2017 Feb 14;12(2):e0171763. doi: 10.1371/journal.pone.0171763 (PMC5308779; doi:10.1371/journal.pone.0171763)
Supplement: S7 Table — (DOCX) [file pone.0171763.s009.docx]

**S7 Table. Gene transcription levels (log_2_FC) of selected genes measured by RNA-seq and qRT-PCR in *D. magna* following 21-d exposure to 2 mg/L of BTR, 5MeBTR and 5ClBTR.**

| Transcript ID | Gene name | Gene symbol |  | BTR | 5MeBTR | 5ClBTR |
| --- | --- | --- | --- | --- | --- | --- |
| TR315\|c0_g1_i2 | endochitinase-like | *cht* | RNA-seq | **5.24^a^** | **-2.62** |  |
|  |  |  | qRT-PCR | 0.46 | -0.20 |  |
| TR10295\|c0_g1_i1 | Cuticular protein 27 | *cp27* | RNA-seq | **5.65** |  |  |
|  |  |  | qRT-PCR | 0.96 |  |  |
| TR1915\|c0_g1_i1 | cuticle protein | *cp* | RNA-seq | **7.04** |  |  |
|  |  |  | qRT-PCR | 0.25 |  |  |
| TR21858\|c0_g1_i6 | chitinase 3 | *cht3* | RNA-seq | **7.08** | **-3.33** |  |
|  |  |  | qRT-PCR | 0.25 | -0.20 |  |
| TR1754\|c0_g1_i1 | Kr-h2 (Krueppel homolog) | *kr-h2* | RNA-seq |  |  | **-7.36** |
|  |  |  | qRT-PCR |  |  | **-1.01** |
| TR21790\|c0_g1_i1 | Apolipoprotein D | *apod* | RNA-seq | **-8.44** |  |  |
|  |  |  | qRT-PCR | **-5.20** |  |  |
| TR6948\|c0_g2_i1 | T-complex protein 1 subunit delta | *cct4* | RNA-seq |  | **-9.56** |  |
|  |  |  | qRT-PCR |  | -0.38 |  |

^a^ Gene transcription values are given as log_2_ (fold change). Significant differential transcription values are indicated in bold (*p*<0.05)
