# Supplementary material for: Transcriptomic, cellular and life-history responses of Daphnia magna chronically exposed to benzotriazoles: Endocrine-disrupting potential and molting effects
Source: PLoS One. 2017 Feb 14;12(2):e0171763. doi: 10.1371/journal.pone.0171763 (PMC5308779; doi:10.1371/journal.pone.0171763)
Supplement: S8 Table — Genes were selected based on their differential transcription measured by RNA-seq (genes in bold) and for their role in endocrine-mediated molting processes in Daphnia. Acronym definition can be found in S6 Table. (DOCX) [file pone.0171763.s010.docx]

**S8 Table. Gene transcription values (log_2_FC) measured by qRT-PCR in *D. magna* exposed for 21-d to 2 and 2000 µg/L of BTR, 5MeBTR and 5ClBTR.** Genes were selected based on their differential transcription measured by RNA-seq (genes in bold) and for their role in endocrine-mediated molting processes in Daphnia. Acronym definition can be found in S6 Table.

|  | BTR |  | 5MeBTR | | 5ClBTR | |
| --- | --- | --- | --- | --- | --- | --- |
|  | 2 | 2000 | 2 | 2000 | 2 | 2000 |
| cuticle |  |  |  |  |  |  |
| ***cprr2*** | -9,01^a^ | 0,00 | 0,81 | 0,69 | -0,03 | 0,84 |
| ***cp27*** | 0,09 | 0,96 | 0,79 | **1,78** | -1,23 | -1,60 |
| ***cp*** | -6,66 | 0,25 | -1,27 | 0,13 | -0,01 | -1,31 |
| ***cht3*** | -1,35 | 0,25 | 0,59 | -0,78 | -0,48 | -0,23 |
| ***cht*** | -2,05 | 0,46 | 0,30 | -0,20 | -0,27 | -0,60 |
| 20HE-mediated | |  |  |  |  |  |
| *ecr* | -0,08 | 0,24 | -0,22 | -0,37 | -0,01 | -0,53 |
| *usp* | -0,14 | -0,16 | -0,20 | **-0,41** | 0,06 | -0,33 |
| *hr3* | -0,30 | -1,60 | -1,60 | -2,81 | -0,13 | -0,11 |
| *ftz-f1* | 1,52 | 2,64 | -1,00 | -0,56 | 0,75 | 0,68 |
| *18a1* | 0,39 | 1,95 | **-1,30** | **-1,52** | -0,31 | 0,65 |
| MF-mediated | |  |  |  |  |  |
| *famt* | -0,03 | -0,83 | -0,51 | -0,90 | -0,49 | 0,01 |
| *met* | -0,47 | **-0,69** | **-0,54** | **-0,73** | -0,20 | **-0,69** |
| *src* | -0,11 | -0,29 | -0,36 | **-0,89** | -0,18 | -0,24 |
| *kr-h1* | -0,14 | -0,12 | -0,27 | **-0,70** | -0,73 | **-1,01** |

^a^ Gene transcription values are given as log2 (fold change). Significant differential transcription values are indicated in bold (*p*<0.05)
